# Real-world cost-effectiveness of rivaroxaban and apixaban vs VKA in stroke prevention in non-valvular atrial fibrillation in the UK

**DOI:** 10.1080/20016689.2020.1782164

**Published:** 2020-06-25

**Authors:** Kevin Bowrin, Jean-Baptiste Briere, Pierre Levy, Aurélie Millier, Jean Tardu, Mondher Toumi

**Affiliations:** a Bayer Plc, Reading, UK; b Bayer AG, Berlin, Germany; c Université Paris-Dauphine, PSL Research University, LEDa-LEGOS, Paris, France; d Creativ-Ceutical, Paris, France; e Aix-Marseille University, Marseille, France

**Keywords:** Anticoagulants, atrial fibrillation, cost-effectiveness, economic, real-world evidence, stroke prevention

## Abstract

**Background:**

Morbidity and mortality associated with non-valvular atrial fibrillation (NVAF) imposes a substantial economic burden on the UK healthcare system.

**Objectives:**

An existing Markov model was adapted to assess the real-world cost-effectiveness of rivaroxaban and apixaban, each compared with a vitamin K antagonist (VKA), for stroke prevention in patients with NVAF from the National Health Service (NHS) and personal and social services (PSS) perspective.

**Methods:**

The model considered a cycle length of 3 months over a lifetime horizon. All inputs were drawn from real-world evidence (RWE): baseline patient characteristics, clinical event and persistence rates, treatment effect (meta-analysis of RWE studies), utility values and resource use. Deterministic and probabilistic sensitivity analyses were performed.

**Results:**

The incremental cost per quality-adjusted life year was £14,437 for rivaroxaban, and £20,101 for apixaban, compared with VKA. The probabilities to be cost-effective compared with VKA were 90% and 81%, respectively for rivaroxaban and apixaban, considering a £20,000 threshold. In both comparisons, the results were most sensitive to clinical event rates.

**Conclusions:**

These results suggest that rivaroxaban and apixaban are cost-effective vs VKA, based on RWE, considering a £20,000 threshold, from the NHS and PSS perspective in the UK for stroke prevention in patients with NVAF. This economic evaluation may provide valuable information for decision-makers, in a context where RWE is more accessible and its value more acknowledged.

## Introduction

As part of the core reimbursement dossier, Health Technology Assessment bodies require both the clinical and economic evaluations of new technologies [1–4]. Randomized clinical trials (RCTs) are currently the main source of efficacy data required in cost-effectiveness models that support economic evaluations. Although models using RCT data are widely accepted, according to the hierarchy of evidence, there is increasing attention on the use of real-world evidence (RWE) to populate effectiveness data in cost-effectiveness models [2,4,5].

RCTs and RWE differ in several aspects. First, RCT populations are selected based on inclusion/exclusion criteria, and are treated under highly regulated conditions, while RWE reflects the situation in a real-world patient population. Second, RCT sample size is limited whereas RWE can be based on a large sample size. Third, the timeframe of RCTs is usually short, with only a selection of outcomes, and long-term outcomes are often not available. RWE can provide results on a broader range of outcomes than are typically observed in RCTs, and can bestow more longitudinal insights whilst RCTs are usually focused on first-time event data collection [1,4]. RWE is also associated with several challenges because treatment assignment is non-random, the estimated effects of treatment on outcomes are subject to bias in attributing causality and estimating the relative effects of a treatment. Although RWE may be considered as methodologically weaker than RCTs, it is now widely acknowledged that these studies can support and further extend efficacy findings from RCTs to large patient populations as encountered in real-world clinical practice [6]. Based on these considerations, the addition of RWE can provide more realistic estimates of cost-effectiveness, based on how the drug is being used in clinical practice and the associated costs [2].

This paper aims to provide an illustration of a cost-effectiveness model considering RWE, using as an example an economic evaluation of oral anticoagulation treatment for the prevention of stroke in patients with non-valvular atrial fibrillation (NVAF), in clinical practice in the UK.

Atrial fibrillation (AF) is a condition characterized by an abnormal heart rhythm resulting from irregular electrical signals. It is the most prevalent cardiac arrhythmia and can manifest as chest pain, dyspnoea, palpitation and dizziness or loss of consciousness [7]. Associated morbidity and mortality imposes a substantial economic burden on the healthcare system [8,9].

Oral anticoagulation has been established as a cornerstone of the management of patients with NVAF and has been shown to reduce the incidence of stroke and mortality [10]. Oral anticoagulation treatments include vitamin K antagonists (VKAs), or non-vitamin K antagonist oral anticoagulants (NOACs), classified as direct thrombin inhibitors (dabigatran etexilate) and factor Xa inhibitors (rivaroxaban, apixaban and edoxaban) [11–14]. During their respective National Institute for Health and Care Excellence (NICE) evaluations, all the NOACs were found to be cost-effective vs VKA [11–14]. The present analysis is an update of the model submitted to NICE for rivaroxaban vs VKA [12], considering RWE for all inputs. The same analysis is replicated for apixaban vs VKA as an additional comparison. This RWE economic analysis may provide valuable information because although VKA is known to work well in clinical trial settings, it is likely that the numerous food and drug interactions and associated monitoring burden impact the real-life effectiveness compared with NOACs.

## Methods

### Model overview

An existing Markov model was updated to assess the comparative costs and outcomes of rivaroxaban and apixaban, each compared with VKA, for the first-line treatment of adult patients with NVAF and with more than one risk factor for stroke [12]. This analysis considered the National Health Service (NHS) and personal and social services (PSS) perspective, over a lifetime horizon (30 years of simulation).

**Figure 1.**
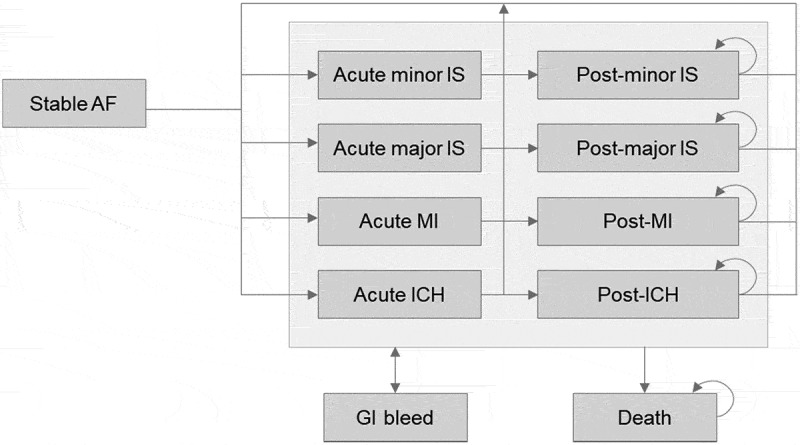
Model diagram.

AF, atrial fibrillation; GI, gastrointestinal bleed; ICH, intracranial haemorrhage; IS, ischaemic stroke; MI, myocardial infarction

As described elsewhere [15], the model comprises a series of health states based on potential complications of NVAF (stable AF, acute and post-major ischaemic stroke [IS], acute and post-minor IS, acute and post-myocardial infarction [MI], acute and post-intracranial haemorrhage [ICH] and gastrointestinal [GI] bleed), and the absorbing state of death (Figure 1). Patients transition through the model in cycles of 3 months, accumulating quality-adjusted life years (QALYs) associated with each health state, together with the costs of treatment, events and subsequent monitoring.

After initial treatment allocation, patients can discontinue their initial treatment, switch from rivaroxaban (or from apixaban) to VKA, switch from VKA to another VKA, or stop treatment, i.e. switch from any treatment to no treatment. Whatever the treatment, long-term consequences of events (first and subsequent) were considered until death.

The model outcomes included the number of IS, the number of MI and the number of bleeds (ICH and GI), as well as the total QALYs, the total life-years (LY) gained, the total costs and the incremental cost per QALY or per LY gained.

### Model input parameters

All inputs are presented in Table 1.

**Table 1.** Model inputs.

	Value	Range used in DSA	Distribution used in PSA	Source
**3-month probabilities (VKA arm)**				
Minor IS	0.151%	[0.146%; 0.155%]	Beta (Alpha = 4539; Beta = 3,005,582)	Weighted average of event rates identified in Briere et al. [26] & Hylek et al. [27]
Major IS	0.217%	[0.211%; 0.223%]	Beta (Alpha = 4536; Beta = 2,085,855)	
MI	0.193%	[0.181%; 0.205%]	Beta (Alpha = 1037; Beta = 536,223)	
GI bleed	0.406%	[0.395%; 0.417%]	Beta (Alpha = 5469; Beta = 1,341,752)	
ICH	0.199%	[0.190%; 0.208%]	Beta (Alpha = 1778; Beta = 891,500)	
Discontinuation				
0–3 months	6.10%	[5.61%; 6.59%]	Beta (Alpha = 567; Beta = 8735)	Johnson et al. [30]
3–6 months	7.35%	[6.82%; 7.88%]	Beta (Alpha = 684; Beta = 8618)	
6–12 months	5.44%	[4.97%; 5.90%]	Beta (Alpha = 506; Beta = 8796)	
12+ months	4.02%	[3.630%; 4.41%]	Beta (Alpha = 390; Beta = 9317)	
**Proportion of switch**				
From VKA to VKA	25.80%	[21.93%; 29.67%]	Beta (Alpha = 126; Beta = 364)	Johnson et al. [30]
From rivaroxaban to VKA	23.20%	[19.72%; 26.68%]	Beta (Alpha = 131; Beta = 433)	
From apixaban to VKA	36.70%	[31.20%; 42.21%]	Beta (Alpha = 108; Beta = 186)	
**Hazard ratio (rivaroxaban arm)**				
Minor IS	0.83	[0.75; 0.93]	Not applicable*	HRs from Coleman et al. [29]
Major IS				
MI	0.96	[0.80; 1.14]		
GI bleed	1.22	[1.12; 1.33]		
ICH	0.68	[0.52; 0.90]		
Discontinuation	0.62	[0.60; 0.65]		
**3-month probabilities (rivaroxaban arm)**				
Minor IS	0.125%	[0.113%; 0.140%]	Beta (Alpha = 414; Beta = 330,342)	Calculation from HRs from Coleman et al. [29]
Major IS	0.180%	[0.163%; 0.202%]	Beta (Alpha = 414; Beta = 229,307)	
MI	0.185%	[0.154%; 0.220%]	Beta (Alpha = 138; Beta = 74,453)	
GI bleed	0.495%	[0.455%; 0.540%]	Beta (Alpha = 5469; Beta = 1,341,752)	
ICH	0.135%	[0.104%; 0.179%]	Beta (Alpha = 1778; Beta = 891,500)	
Discontinuation				
0–3 months	3.78%	[3.66%; 3.97%]	Beta (Alpha = 3552; Beta = 90,370)	Calculation from HRs from Coleman et al. [29] and Johnson et al. [30]
3–6 months	4.56%	[4.41%; 4.78%]	Beta (Alpha = 3524; Beta = 73,816)	
6–12 months	3.37%	[3.26%; 3.53%]	Beta (Alpha = 3567; Beta = 102,297)	
12+ months	2.49%	[2.41%; 2.61%]	Beta (Alpha = 3600; Beta = 140,829)	
**Hazard ratio (apixaban arm)**				
Minor IS	1.01	[0.87; 1.17]	Not applicable*	HRs from Coleman et al. [29]
Major IS				
MI	1.00	NA		
GI bleed	0.52	[0.38; 0.70]		
ICH	0.41	[0.28; 0.60]		
Discontinuation	1.08	[0.81; 1.45]		
**3-month probabilities (apixaban arm)**				
Minor IS	0.152%	[0.131%; 0.176%]	Beta (Alpha = 200; Beta = 131,289)	Calculation from HRs from Coleman et al. [29]
Major IS	0.219%	[0.189%; 0.254%]	Beta (Alpha = 200; Beta = 91,113)	
MI	0.193%	[0.181%; 0.205%]	Beta (Alpha = 1037; Beta = 536,223)	
GI bleed	0.211%	[0.154%; 0.284%]	Beta (Alpha = 53; Beta = 25,015)	
ICH	0.082%	[0.056%; 0.119%]	Beta (Alpha = 38; Beta = 46,742)	
Discontinuation				
0–3 months	6.59%	[4.94%; 8.85%]	Beta (Alpha = 57; Beta = 813)	Calculation from HRs from Coleman et al. [29] and Johnson et al. [30]
3–6 months	7.94%	[5.95%; 10.65%]	Beta (Alpha = 57; Beta = 656)	
6–12 months	5.87%	[4.40%; 7.88%]	Beta (Alpha = 58; Beta = 927)	
12+ months	4.34%	[3.26%; 5.83%]	Beta (Alpha = 59; Beta = 1295)	
**In-hospitalisation mortality rates per clinical event in model**				
Minor IS	0.00%	-	-	Assumption
Post-minor IS	0.00%	-	-	
Major IS	25.57%	[25.09%; 26.05%]	Beta (Alpha = 7996; Beta = 23,276)	Seminog et al. [16]
Post-major IS	8.12%	[7.35%; 8.92%]	Beta (Alpha = 390; Beta = 4410)	Lip et al. [17]
MI	24.67%	[23.79%; 25.55%]	Beta (Alpha = 2257; Beta = 6893)	Smolina et al. [18]
Post-MI	8.24%	[7.17%; 9.34%]	Beta (Alpha = 211; Beta = 2347)	Lip et al. [17]
ICH	28.50%	[24.23%; 32.78%]	Beta (Alpha = 122; Beta = 306)	Nuffield Trust [19]
Post-ICH	14.11%	[11.85%; 16.57%]	Beta (Alpha = 128; Beta = 781)	Lip et al. [17]
GI bleed	14.63%	[13.74%; 15.52%]	Beta (Alpha = 882; Beta = 5147)	Button et al. [20]
**Utility values**				
Stable AF	0.73	[0.71; 0.75]	Beta (Alpha = 1594; Beta = 589)	Sullivan et al. [32]
Minor IS	0.73	[0.55; 0.91]	Beta (Alpha = 45; Beta = 17)	Luengo-Fernandez et al. [34]
Major IS	0.41	[0.31; 0.51]	Beta (Alpha = 100; Beta = 144)	Luengo-Fernandez et al. [34]
Post-minor IS	0.76	[0.57; 0.95]	Beta (Alpha = 45; Beta = 17)	Luengo-Fernandez et al. [34]
Post-major IS	0.56	[0.42; 0.70]	Beta (Alpha = 75; Beta = 59)	Luengo-Fernandez et al. [34]
MI	0.66	[0.53; 0.79]	Beta (Alpha = 57; Beta = 29)	Pockett et al. [35]
Post-MI	0.73	[0.58; 0.88]	Beta (Alpha = 45; Beta = 17)	Pockett et al. [35]
ICH	0.56	[0.45; 0.67]	Beta (Alpha = 75; Beta = 59)	Luengo-Fernandez et al. [34]
Post-ICH	0.67	[0.54; 0.80]	Beta (Alpha = 56; Beta = 27)	Luengo-Fernandez et al. [34]
GI bleed	0.70	[0.56; 0.84]	Beta (Alpha = 51; Beta = 22)	Sullivan et al. [32]
Utility decrement for warfarin	0.013	Scenario only	Not applicable	Gage et al. [33]
Utility decrement for rivaroxaban and apixaban	0.002	Scenario only	Not applicable	Sullivan et al. [32]
**Resource use and costs (£ 2018)**				
**Treatment daily cost**				
VKA	0.09	Not applicable	Not applicable	Ying Zheng et al. [21] UK, BNF NHS price
Rivaroxaban	1.80	Not applicable	Not applicable	
Apixaban	1.90	Not applicable	Not applicable	
**IS costs**				
Acute treatment (minor)	3,996	[2,997; 4,995]	Gamma (Standard error = 510; Alpha = 61; Beta = 65)	Ying Zheng et al. [21]
Acute treatment (major)	24,659	[18,494; 30,824]	Gamma (Standard error = 3145; Alpha = 61; Beta = 401)	
Monthly follow-up (minor)	209	[131; 304]	Gamma (Standard error = 38; Alpha = 277; Beta = 2)	Luengo-Fernandez et al. [34]
Monthly follow-up (major)	1,332	[672; 2,213]	Gamma (Standard error = 112; Alpha = 140; Beta = 9)	
Rehabilitation	8,083	[6,062; 10,104]	Gamma (Standard error = 1031; Alpha = 61; Beta = 132)	
**MI**				
Acute treatment (one event per cycle)	1,957	[1,468; 2,446]	Gamma (Standard error = 250; Alpha = 61; Beta = 32)	NHS reference cost
Monthly follow-up	217	[189; 246]	Gamma (Standard error = 169; Alpha = 15; Beta = 44)	Danese et al. [22]
**Bleeds**				
Acute treatment for GI bleed (non-ICH)	1,617	[1,212; 2,021]	Gamma (Standard error = 206; Alpha = 61; Beta = 32)	NHS reference cost
Acute treatment – ICH	2,985	[2,239; 3,731]	Gamma (Standard error = 381; Alpha = 61; Beta = 49)	
Monthly follow-up	418	[337; 508]	Gamma (Standard error = 44; Alpha = 830; Beta = 2)	Campbell et al. [23]
Rehabilitation	5,036	[3,777; 6,295]	Gamma (Standard error = 642; Alpha = 61; Beta = 82)	Luengo-Fernandez et al. [34]
**Resource use for rehabilitation**				
% of rehabilitation for major IS	34.8%	[34.1%; 35.5%]	Beta (Alpha = 6928; Beta = 12,980)	Cotté et al. [24]
% of rehabilitation for GI bleed	14.2%	[13.4%; 15.0%]	Beta (Alpha = 1063; Beta = 6421)	
% of rehabilitation for ICH	32.9%	[31.5%; 34.3%]	Beta (Alpha = 1391; Beta = 2836)	

* HRs were not included per se in the PSA, instead, the 3-month probabilities for apixaban and rivaroxaban were included, calculated from the HRs.

Abbreviations: AF, atrial fibrillation; DSA, deterministic sensitivity analysis; GI, gastrointestinal; HR, hazard ratio; ICH, intracranial haemorrhage; IS, ischaemic stroke; MI, myocardial infarction; PSA, probabilistic sensitivity analysis; VKA, vitamin K antagonist.

#### Patient population

To ensure generalizability of the NVAF population in the UK, the model was populated with patient and clinical characteristics drawn from a recent UK database study on AF [25]. Patients entered the model at a mean age of 75 years, 10% had an intermediate CHA_2_DS_2_-VASc score (=1) and 90% had a high CHA_2_DS_2_-VASc score (≥2).

#### Event rates

Transition probabilities of the VKA arm were derived from existing RWE studies providing event rates per patient for IS, MI, ICH and GI bleeds [26]. The proportion of IS that were minor and major was derived from another RWE study [27]. In addition, for minor and major IS, the risk was adjusted by age using results from the RWE Framingham Heart Study, so that with increasing age of the patient cohort, the risk was increased accordingly, this is presented in Table 2 [28]. The treatment effect evidence for rivaroxaban and apixaban was taken from a published meta-analysis, providing hazard ratios (HRs) vs VKA considering RWE in both prevalent and incident populations [29].

**Table 2.** Relative risks for ischaemic stroke by age group [28].

Age group	Relative risk
55–59	0.667
60–64	0.760
65–69	0.854
70–74	1.000
75–79	1.146
80–84	1.281
85–89	1.480
90+	1.719

#### Discontinuation

As the risk of discontinuation is unlikely to be constant over time, the model was updated to capture the evolution of persistence with time [30]. Discontinuation was split into different periods: from initiation to 3 months, from 3 months to 6 months, from 6 months to 1 year and after 1 year. The persistence rates used for VKA were taken from a recent UK study [30]. The comparative treatment effect for rivaroxaban and apixaban vs VKA, was taken from the previously mentioned RWE meta-analysis [29].

#### Mortality

A background mortality rate was applied to all states within the model, as identified from the latest UK life tables available (2016) [31]. A number of health states within the economic model also included a specific mortality-related risk in addition to a background mortality rate, as shown in Table 1.

#### Utility

Utility values were derived from UK studies [32–35]. No utility decrements associated with treatments were considered in the base case analysis.

#### Resource use and costs

The cost categories comprise drug acquisition costs, administration costs, VKA monitoring costs and costs associated with clinical events. All resource use and costs were taken from RWE studies or reference costs, and all costs were inflated to 2018 levels when necessary. NHS reference costs were used where possible, especially for drug acquisition calculations or management costs, such as ICH bleeding and MI management costs, or VKA monitoring costs.

#### Sensitivity analyses

A deterministic sensitivity analysis (DSA) was conducted to test the impact of variations in the parameters included in the model, and a probabilistic sensitivity analysis (PSA) was performed to evaluate the uncertainty of the model parameters on the cost-effectiveness results. In addition, several specific scenarios were considered.

The first scenario considers the inclusion of utility decrements related to treatment: a utility decrement of 0.013 was associated with VKA and a utility decrement of 0.002 was associated with rivaroxaban and apixaban therapy [12,13]. A second scenario considered VKA baseline event rates for incident patients [36], as well as HRs drawn from studies including incident patients only, to account for bleeding events usually occurring in the initial phases of anticoagulant treatment [37].

## Results

Table 3 presents the results of the analyses for rivaroxaban vs VKA, and apixaban vs VKA.

**Table 3.** Model results.

Outcome	VKA	Rivaroxaban		Apixaban		
	Value	Value	Incr. vs VKA	Value		Incr. vs VKA
**Costs (£)**						
Drug acquisition costs	158	3,285	3,127	2,568		2,410
Drug administration costs	1,310	947	−363	908		−402
Event treatment costs	8,421	7,366	−1,055	8,258	−163	
Total costs	9889	11,598	1709	11,734	1846	
**Health benefits**						
Total QALYs	5.96	6.08	0.12	6.06	0.09	
Total LYs	8.25	8.40	0.15	8.37	0.12	
Ischaemic strokes	0.265	0.228	−0.037	0.266	0.001	
Myocardial infarction	0.096	0.088	−0.008	0.97	0.0005	
Bleeds	0.130	0.152	0.023	0.089	−0.041	
Intracranial haemorrhage	0.042	0.036	−0.006	0.027	−0.015	
GI bleeds	0.088	0.117	0.029	0.063	−0.025	
**Incremental costs-effectiveness ratios (£)**						
Incremental cost/QALY			£14,437		£20,101	
Incremental cost/LY			£11,299		£14,912	

Abbreviations: GI, gastrointestinal; QALY, quality-adjusted life year; LY, life year; VKA, vitamin K antagonist.

Patients treated with rivaroxaban experienced incremental gains in both QALYs (0.12) and LYs (0.15) compared with VKA: rivaroxaban was associated with fewer cases of MI (0.088 vs 0.096, i.e. a reduction of 8%), and a lower rate of strokes (0.228 vs 0.265, i.e. a reduction of 14%) and ICH bleeds (0.036 vs 0.042 i.e. a reduction of 15%), but was associated with a higher rate of GI bleeds (0.117 vs 0.088, i.e. an increase of 33%). From the NHS and PSS perspective, the benefits translated into an incremental cost per QALY of £14,437 and an incremental cost per LY of £11,299.

Patients treated with apixaban experienced a lower rate of ICH bleeds (0.027 vs 0.042 i.e. a reduction of 37%) and GI bleeds (0.063 vs 0.088 i.e. a reduction of 29%) compared with VKA, however, no reductions were observed for rates of MI and strokes because the associated HRs were close to, or equal to 1. This translated into incremental gains in both QALYs (0.09) and LYs (0.12) compared with VKA, and into an incremental cost per QALY of £20,101 and an incremental cost per LY of £14,912.

**Figure 2.**
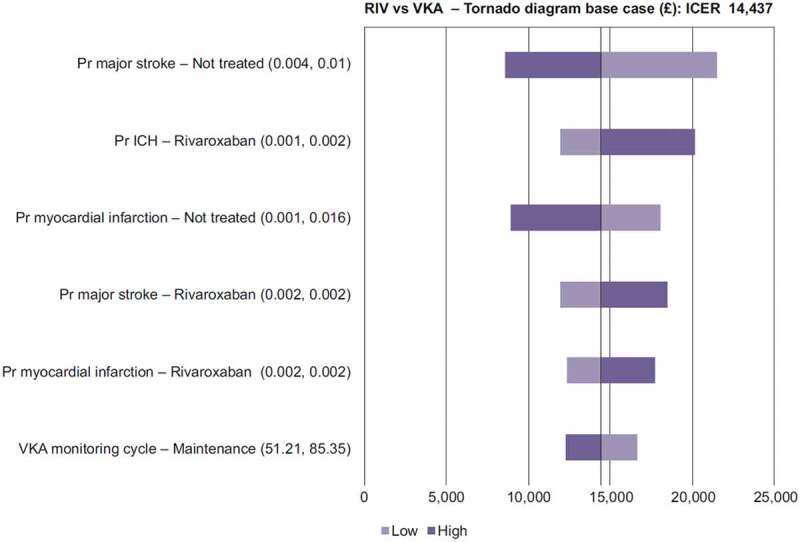
Rivaroxaban tornado diagram.

ICER, incremental cost-effectiveness ratio; ICH, intracranial haemorrhage; Pr, probability; RIV, rivaroxaban; VKA, vitamin K antagonist

**Figure 3.**
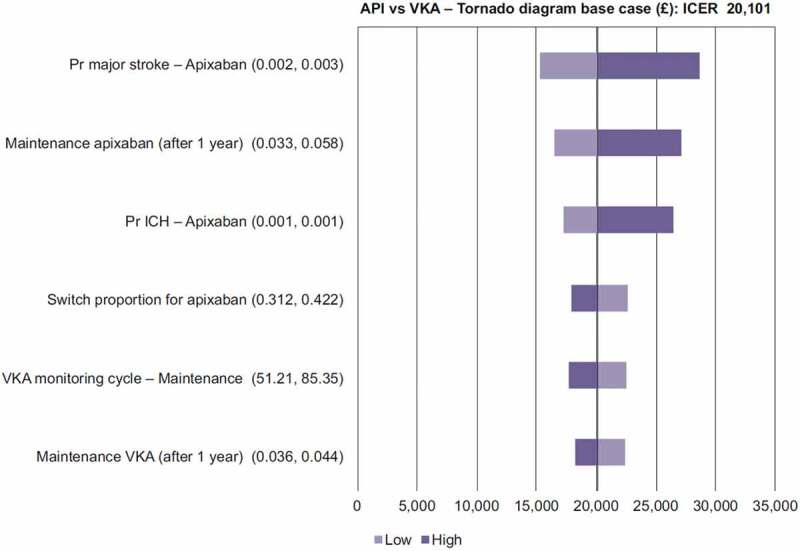
Apixaban tornado diagram.

API, apixaban; ICER, incremental cost-effectiveness ratio; ICH, intracranial haemorrhage; Pr, probability; VKA, vitamin K antagonist.

Figures 2 and 3 show that the results were robust to plausible changes with respect to input parameters, with most parameters having a minimal impact on the results. The main drivers of the incremental cost-effectiveness ratios (ICERs) were different depending on the comparison: for rivaroxaban, the main drivers identified were major stroke probability, ICH probability and MI probability; they were major stroke probability, apixaban maintenance after 1 year and ICH probability for apixaban.

**Figure 4.**
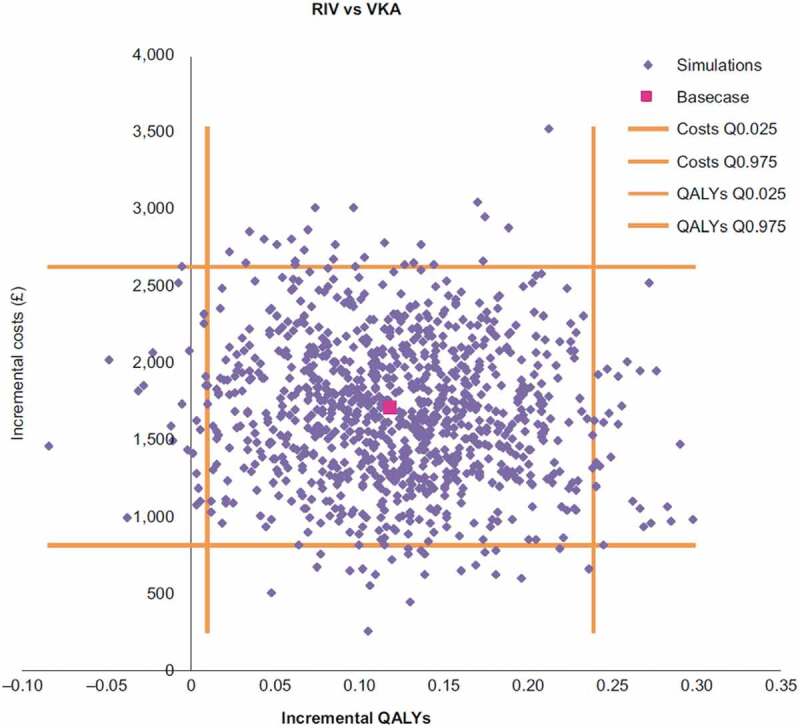
Rivaroxaban incremental cost-effectiveness plane.

QALY, quality-adjusted life years; RIV, rivaroxaban; VKA, vitamin K antagonist

**Figure 5.**
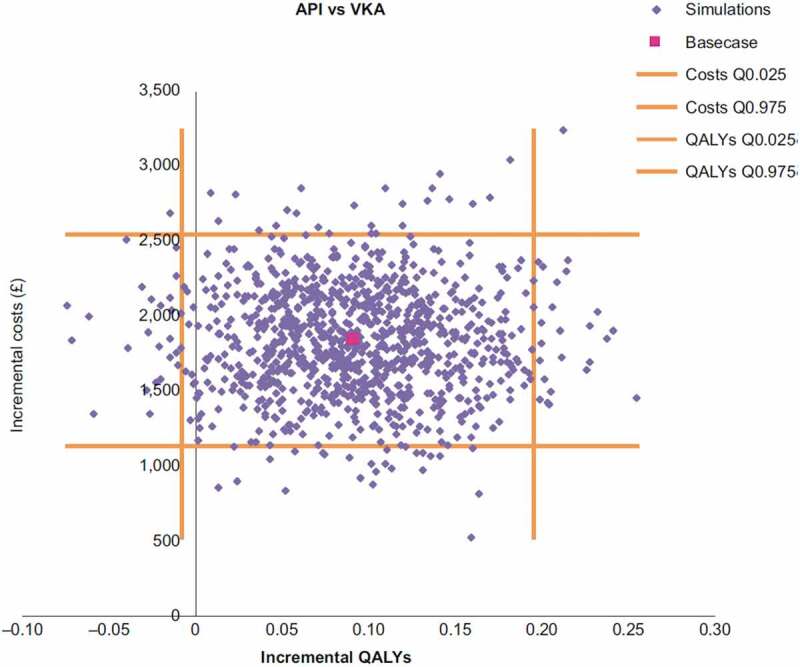
Apixaban incremental cost-effectiveness plane.

API, apixaban; QALY, quality-adjusted life years; VKA, vitamin K antagonist

Cost-effectiveness scatterplots for rivaroxaban vs VKA and for apixaban vs VKA are presented respectively in Figures 4 and 5. The probabilities for each NOAC to be cost-effective compared with VKA considering a £20,000 threshold were 90% and 81%, respectively for rivaroxaban and apixaban.

Several scenarios were conducted. First, applying a utility decrement associated with NOAC and VKA therapy (scenario 1) reduced the ICER to £11,630 per QALY gained for rivaroxaban, and to £15,660 per QALY gained for apixaban. Finally, considering treatment incident studies only from the meta-analysis (scenario 2) reduced the ICER to £13,721 per QALY gained for rivaroxaban, and increased to £25,166 per QALY gained for apixaban.

## Discussion

The use of NOACs for the prevention of stroke in patients with NVAF is an ideal example to test RWE in a cost-effectiveness analysis. RCTs have shown NOACs to be at least as efficacious as VKA for the prevention of stroke or systemic embolism in patients with NVAF [38–40], and numerous regulatory bodies and guidelines worldwide have endorsed the use of NOACs [41].

Existing cost-effectiveness analyses of NOACs have found these treatments to be cost-effective [42–44]. Although the upfront monthly cost of NOACs exceeds that of VKA, the reduction in clinical events such as stroke, associated with NOACs, as well as the avoided costs due to rehabilitation, likely offset the financial burden of these drugs whilst increasing the quality of life for patients. In addition, VKA use requires regular international normalized ratio (INR) monitoring tests to ensure that drug concentrations are within a defined, narrow therapeutic range; these direct costs (laboratory tests) are also offset with NOAC use [45].

A review of the UK NICE dossiers for the four available NOACs and of their associated appraisals [11–14] revealed that all reviewed NOACs demonstrated a treatment benefit in stroke reduction but that several elements would benefit from RWE. First, key cost-effectiveness drivers included several factors that could potentially vary in RWE data. These included the discontinuation rate, cost of INR monitoring visits and patient baseline age. In addition, considerable variation between different centres and settings, depending on the patient group, were also identified. Also, the generalizability of clinical evidence to patients diagnosed with AF in the national population for modelling was a key element of uncertainty. The integration of RWE into cost-effectiveness analyses of NOACs, therefore, provides an additional level of information that can be used to evaluate the clinical and economic value of these medicines.

The model structure of this cost-effectiveness analysis was based on the previous submission of rivaroxaban to NICE [12]; no major criticism was raised by the evidence review group at that time. Nevertheless, in order to reflect the context of RWE in stroke prevention in AF, several structural adjustments were made. First, the approach used to model persistence was improved with inclusion of more granularity. Second, possibilities of treatment switches were updated, to ensure more generalizability.

This model is considered to be a RWE cost-effectiveness model as all the evidence came from RWE. The initial population is based on a RWE study, to reflect the characteristics of patients with AF in the UK. Patients progress between health states according to transition probabilities derived from RWE: RWE is considered both for event rates for VKA, but also for treatment effects for rivaroxaban and apixaban. The treatment effects were taken from RWE meta-analyses performed for each drug separately vs VKA [29], as well as costs and utilities. Of note, all key cost-effectiveness drivers of economic models submitted to NICE, including discontinuation rates, cost of INR monitoring visits and patient baseline age were drawn for RWE sources in the current model.

The results of the present analysis are consistent with the NICE evaluations [12,13], where both rivaroxaban and apixaban were found to be cost-effective compared with VKA. A more precise comparison cannot be conducted because the NICE did not report any definite ICER in any of the reports: the ICER of rivaroxaban ranged between £2870 and £29,500 per QALY gained [12], and no conclusive ICER was given in the apixaban report [13]. Although the conclusions remain the same using RWE, the ICER for rivaroxaban vs VKA was found to be lower than the ICER for apixaban vs VKA in the present analysis; the main driver for this difference was the incremental QALYs, which we estimate to have increased for rivaroxaban, but were estimated to be reduced for apixaban, resulting from the use of the RWE meta-analysis to populate treatment effect. In this RWE meta-analysis, the HRs for stroke and MI with apixaban were less favourable than the HRs of the RCT, whereas the opposite was observed for rivaroxaban.

When compared with VKA, while apixaban was associated with a decrease in the rate of GI bleeds, rivaroxaban was associated with fewer strokes. Because of the high management cost of this event, a strong impact on ICER was observed. The ICER vs VKA was below £20,000, suggesting rivaroxaban is cost-effective from the NHS and PSS perspective. For apixaban, the ICER was slightly above the threshold, however, more than 80% of the simulations showed apixaban to be cost-effective, suggesting these results should be interpreted with caution.

This confirms previous results submitted to NICE [12,13], but also results published later, in the UK in particular [46], but also in France [15]. This latter paper used a similar approach for rivaroxaban, considering only RWE inputs (characteristics, clinical event rates, treatment effect, discontinuation, switch rates, utility, resource use and unit costs for all events), and led to a similar conclusion.

Although efforts were made to ensure the best structure and approach were used, considering clinician and health economist experts’ validation, several limitations should be considered. First, as in all models, this approach should be considered as a simplification of what is expected in real life. A number of assumptions are used to reflect adequately the long-term consequences of NVAF. Second, the use of multiple RWE studies included in the meta-analyses to estimate all treatment effect parameters raises the question of uncertainty, linked to the heterogeneity of RWE studies. The influence of several factors was investigated by repeating the meta-analysis of the RWE studies using several scenarios, by varying population characteristics or type of statistical adjustment used to control bias and adjusting for the quality of studies included [36]. Some variations were noted, but overall, the results were robust and associated with a high level of external validity. Another limitation associated with other inputs, whether economic, epidemiological or related to utility, should be mentioned: although all studies used to populate the model inputs were drawn from RWE, a UK source could not be retrieved for all. Finally, it should be added that this analysis does not present comparisons of dabigatran or of edoxaban vs VKA.

## Conclusion

This RWE economic analysis demonstrated that rivaroxaban is cost-effective vs VKA, from the NHS and PSS perspective, with an incremental cost per QALY of £14,437. Because of a lower efficacy in terms of MI and IS rate reduction, the ICER for apixaban vs VKA was £20,101 per QALY, slightly above the UK threshold of £20,000 per QALY; however, the sensitivity analyses showed a probability to be cost-effective exceeding 80%. Although several limitations are associated with this study, particularly due to the heterogeneity of RWE studies, these results are considered stable, providing valuable information to decision-makers, in a context where RWE is more accessible, and its value more acknowledged.
